# Aryl Hydrocarbon Receptor Ligands Inhibit IGF-II and Adipokine Stimulated Breast Cancer Cell Proliferation

**DOI:** 10.1155/2013/104850

**Published:** 2013-09-23

**Authors:** Travis B. Salisbury, Gary Z. Morris, Justin K. Tomblin, Ateeq R. Chaudhry, Carla R. Cook, Nalini Santanam

**Affiliations:** ^1^Department of Pharmacology, Physiology and Toxicology, Joan C. Edwards School of Medicine, Marshall University, 1 John Marshall Drive, Huntington, WV 25755, USA; ^2^Department of Science and Mathematics, Glenville State College, WV 26351, USA

## Abstract

Obesity increases human cancer risk and the risk for cancer recurrence. Adipocytes secrete paracrine factors termed adipokines that stimulate signaling in cancer cells that induce proliferation. The aryl hydrocarbon receptor (AHR) is a ligand-activated transcription factor that plays roles in tumorigenesis, is regulated by exogenous lipophilic chemicals, and has been explored as a therapeutic target for cancer therapy. Whether exogenous AHR ligands modulate adipokine stimulated breast cancer cell proliferation has not been investigated. We provide evidence that adipocytes secrete insulin-like growth factor 2 (IGF-2) at levels that stimulate the proliferation of human estrogen receptor (ER) positive breast cancer cells. Using highly specific AHR ligands and AHR short interfering RNA (AHR-siRNA), we show that specific ligand-activated AHR inhibits adipocyte secretome and IGF-2-stimulated breast cancer cell proliferation. We also report that a highly specific AHR agonist significantly (*P* < 0.05) inhibits the expression of E2F1, CCND1 (known as Cyclin D1), MYB, SRC, JAK2, and JUND in breast cancer cells. Collectively, these data suggest that drugs that target the AHR may be useful for treating cancer in human obesity.

## 1. Introduction

Human obesity is common and has been linked with increases in breast cancer risk and breast cancer recurrence [1–3]. Although the underlying links between obesity and cancer are not completely clear, adipocytes themselves are postulated to play a role [1–9]. Adipocytes secrete multiple paracrine factors termed adipokines that stimulate signaling in human cancer cells that stimulate proliferation [4–7, 9]. Specific adipokines that stimulate the proliferation of human estrogen receptor (ER) positive breast cancer cells are leptin, collagen VI, and members of the insulin-like growth factor (IGF) family of proteins [4–6, 8, 9]. In ER positive breast cancer cells, leptin through its cognate membrane spanning cytokine leptin receptor activates the JAK/STAT pathway [6, 10]. Collagen VI by activating the NG2/chondroitin sulfate proteoglycan receptor activates AKT and *β*-catenin signaling [4, 5]. IGF-1 and IGF-2 signal through specific cell surface tyrosine kinase receptors, IGF-1 receptor (IGF-1R) and insulin receptor isoform A (IR-A)), that are highly expressed on human ER expressing breast cancer cells [11, 12]. The critical pathway by which IGF-1 and IGF-2 stimulate breast cancer cell proliferation is the PI3K pathway that leads to increases in AKT activity [11, 12]. Leptin, collagen VI, and IGF proteins have all been reported to stimulate increases in the transcription and expression of CCND1 (also known as Cyclin D1) in ER expressing breast cancer cells [4–6, 12]. CCND1 is a regulatory protein that activates the cell cycle, increases cell proliferation, and has been implicated as a promoter of breast tumorigenesis [13]. New drugs and drug targets that inhibit adipokine stimulated breast cancer cell proliferation could be particularly relevant to reducing the higher rates of breast cancer risk and breast cancer recurrence that are observed in obese women compared to women of normal weight. However, there are currently no specific therapies for reducing breast cancer risk and recurrence in obesity.

The aryl hydrocarbon receptor (AHR) is a ligand-activated transcription factor that has been explored as a therapeutic target for cancer [14, 15]. Ligand-activated AHR inhibits the growth of some human cancers cell lines [14, 15]. The AHR is stimulated by lipophilic chemicals that function as AHR agonists including the environmental toxicant 2,3,7,8 tetrachlorodibenzo-p-dioxin (TCDD) and the experimental drug 3-(3,5-dimethyl-1H-pyrrol-2-yl methylene)-1,3-dihydro-indol-2-one (SU5416) [14, 16]. In the absence of an exogenous AHR agonist, the AHR is located in the cytoplasm bound to p23, HSP90, and XAP2 chaperon proteins [17]. Upon activation by an agonist, the AHR dissociates from p23, HSP90, and XAP2, translocates into the nucleus, and stimulates transcription by binding to sequence specific response elements termed AHR response elements (AHRE) in enhancers of genes that are stimulated by AHR ligands [17]. CYP1A1 is a prototypical AHR regulated gene target that has been used to study AHR signaling [17]. Whether specific ligand-activated AHR inhibits adipokine stimulated breast cancer cell proliferation and the potential mechanisms by which this could occur have not been investigated.

Obese women with ER positive breast tumors have worse clinical outcomes and have a higher risk for breast cancer recurrence than obese women with ER negative breast tumors [18]. This suggests that ER positive breast cancer cells could be more sensitive to the proliferative effects of mitogenic adipokines than ER negative breast cancer cells. The human ER positive MCF-7 breast cancer cell line has been used extensively as a model to investigate mitogenic adipokine signaling in human breast cancer cells and MCF-7 cells, express leptin, collagen VI, and IGF receptors [4–6, 12]. The human T-47D breast cancer cell line expresses ER and IGF receptors, and fewer reports have used this cell line to investigate adipokine signaling in human breast cancer cells [14]. Given these prior reports, the purpose of this study was to examine the possibility that specific ligand-activated AHR inhibits mitogenic adipokine signaling in human MCF-7 breast cancer cells and to provide preliminary insights into the mechanism by which this occurs. Primary findings were also validated in T-47D cells.

## 2. Material and Methods

### 2.1. Conditioned Media

A previously published standard adipocyte differentiation protocol was used to differentiate murine 3T3-L1 preadipocyte fibroblasts into fully differentiated adipocytes [4]. Specifically, confluent 3T3-L1 fibroblasts were treated with Dulbecco's Modified Eagle Medium (DMEM), 10% FBS, 160 nM insulin, 250 nM dexamethasone, and 0.5 mM 3-isobutyl-1-methylxanthine (IBMX)) for 3 days, followed by 10% fetal bovine serum (FBS) and 160 nM insulin for 3 days and then DMEM 10% FBS for an additional 6 days, with a medium change every three days. In order to examine the effects of adipocyte secreted adipokines in the absence of confounding factors in FBS, medium was removed from fully differentiated adipocytes, followed by rinsing twice with phosphate buffered saline (PBS), and adipocytes were then incubated in phenol red-free, serum-free DMEM for an additional 24 hr. This serum-free adipocyte conditioned medium (adipo-CM) was centrifuged and stored at −80°C prior to being applied to breast cancer cells in cell culture. Phenol red-free, serum-free DMEM conditioned by 3T3-L1 fibroblasts for 24 hrs was also isolated. Fibroblast conditioned medium (fibro-CM) was applied to breast cancer cells as a control media not conditioned by an adipocyte. DMEM, FBS, P/S, and PBS were purchased from Thermo Fisher Scientific (HyClone Labs, Logan, UT). IBMX, insulin, and dexamethasone were purchased from Sigma-Aldrich (St. Louis, MO).

### 2.2. Breast Cancer Cell Growth Experiments

Prior to specific growth experiments, MCF-7 and T-47D cells (ATCC, (Manassas, VA) were maintained in DMEM, 10% FBS, and 1% penicillin/streptomycin (P/S). To explore whether adipo-CM stimulated cancer proliferation more than fibro-CM, phenol red-free, serum-free DMEM (unconditioned medium), fibro-CM, or adipo-CM was applied to MCF-7 or T-47D cells for three days in culture, after which cells were collected and total live cell number was determined using a hemocytometer and manual cell counting. Preliminary experiments were conducted to determine the optimal dose of TCDD to use in proliferation experiments. In our preliminary experiments, we found that MCF-7 cells were more sensitive to the antiproliferative effects of TCDD than, T-47D cells (data not shown *n* = 3). Thus, in all remaining experiments, MCF-7 cells were treated with 10 nM TCDD and T-47D cells were treated with 100 nM TCDD. The 100 nM dose of SU5416 was selected based on our preliminary data showing that SU5416 at this dose is a strong AHR agonist based on its ability to stimulate increases in CYP1A1 gene expression (data not shown; *n* = 3). Fibro-CM or adipo-CM supplemented with DMSO vehicle, TCDD, or SU5416 was applied to overnight serum-starved (phenol red-free DMEM) MCF-7 or T-47D cells for three days in culture, after which cells were collected and trypan blue and manual cell counting were used to determine live cell number. In other experiments, overnight serum-starved (phenol red-free DMEM) MCF-7 or T-47D cells were stimulated with PBS vehicle or IGF-2 (100 ng/mL; R & D Systems) supplemented with DMSO vehicle, TCDD, or SU5416 for three days in culture, after which cells were collected and live cell was determined with trypan blue. The IGF-2 dose was based on the work of Worster et al. showing that IGF-1 (100 ng/mL) induced the proliferation of human breast epithelial cells [19].

### 2.3. SiRNA Experiments

In order to show that TCDD and SU5416 inhibition of IGF-2 requires the AHR, breast cancer cells were plated in DMEM, 10% FBS, and P/S (80,000 cells per well of a 6-well plate) for 24 hr, then transiently transfected with 50 nM of a single short interfering RNA (siRNA) that specifically targets the AHR (AHR-siRNA) or with a nontargeting control siRNA (con-siRNA) with 2 *μ*L of DharmaFECT reagent 1 for 24 hr. Following removal of transient transfection reagent, cells were serum-starved overnight in phenol red-free DMEM and then treated with IGF-2 (100 ng/mL) supplemented with DMSO vehicle or TCDD (MCF-7 (10 nM), T-47D (100 nM)) or SU5416 (MCF-7 100 nM) for three days in culture, after which cells were collected and trypan blue was used to determine the number of live cells. Con-siRNA (cat number D-001810-01-20), AHR-siRNA (J-004990-05), and DharmaFECT transfection reagent number 1 were purchased from Thermo Scientific, Dharmacon. DMSO and SU5416 were purchased from Sigma-Aldrich (St. Louis, MO). TCDD was purchased from Cambridge Isotopes Laboratory (Andover, MA) (cat number ED-901-B).

### 2.4. Western Blot Experiments

To validate AHR-siRNA mediated knockdown of the AHR, breast cancer cells (300,000 cells per 35 mm plate) were transfected with con-siRNA or AHR-siRNA for 36 hr, followed by isolation of total cellular extract in 250 *μ*L of 2X sample lysis buffer (Bio-RAD; cat number 161-0737). Total cellular extract (~12.5 *μ*g of protein) was subjected to SDS PAGE in Mini-PROTEAN TGX 4–12% Precast Gels (Bio-Rad; Hercules, CA) and transferred to polyvinylidene difluoride (PVDF) membranes (Bio-Rad; Hercules, CA). Membranes were blocked in PBS, 0.05% Tween 20 (PBS-T), and 5% (wt/vol) low-fat powdered milk for 1 hr, followed by overnight incubation at 4°C with rocking with an appropriate primary antibody. Membranes were rinsed five times (five minutes each wash) with PBS-T and then incubated with an appropriate HRP-labeled secondary antibody (Thermo Scientific, Pierce) for 1 h, followed by rinsing five times (five minutes each wash) in PBS-T followed by the application of enhanced chemiluminescent substrate (Thermo Scientific, Millipore) and exposure to X-ray film (Midwest Scientific). Equal protein loading was confirmed by glyceraldehyde 3-phosphate dehydrogenase (abbreviated as GAPDH) western blots. AHR antibody was purchased from Santa Cruz Biotechnology, cat number: sc-5579, and diluted 1 : 5,000 in PBS, 0.01% tween-20, and 5% powdered milk. GAPDH was purchased from Millipore, cat #: MAB374, and diluted 1 : 20,000 dilution in PBS, .01% tween-20, and 5% powdered milk.

### 2.5. RT-qPCR Experiments

A RT^2^ Profiler PCR Array (PAHS-502ZC) (Superarray Bioscience Corporation, Qiagen) was used to compare gene expression between MCF-7 cells treated with IGF-2 (100 ng/mL) plus DMSO vehicle and MCF-7 cells treated with IGF-2 (100 ng/mL) plus TCDD (10 nM) for 48 hrs. The PAHS-502ZC PCR array allows for the integration of 84 genes that are prooncogenes or tumor suppressors and thus are key genes in tumorigenesis. Treatments were stopped, total RNA was isolated (RNeasy Mini Kit, Qiagen), and 1 *μ*g of RNA was converted into cDNA (RT^2^ First Strand Kit, Qiagen). cDNA was combined with RT^2^ SYBER Green ROX qPCR Master Mix (Qiagen), and changes in gene expression were analyzed by RT^2^ Profiler PCR Array (PAHS-502ZC). Statistically significantly differentially expressed genes between groups were calculated by RT^2^ Profiler PCR Array Data Analysis software package that calculates ΔΔ CT calculated fold changes and uses Student's *t*-test to calculate two-tail, equal variance *P* values. Experiments were performed 3 times (*n* = 3).

### 2.6. IGF-2 Blocking Antibody Experiments

In order to explore whether adipocytes secreted levels of IGF-2 are sufficient to stimulate the proliferation of breast cancer cells, a control mouse IgG (5 *μ*g/mL) (R & D systems, cat number MAB002) or human IGF-2 blocking antibody (5 *μ*g/mL) (R & D Systems, cat number MAB292) was added to adipo-CM prior to being added to human breast cancer cells for three days in culture, after which total live cell number was determined using trypan blue. This particular IGF-II blocking antibody was selected because it has been shown to specifically neutralize human IGF-2 in MCF-7 proliferation assays and exhibits 100% cross-reactivity with mouse IGF-2 (R & D Systems product sheet, cat number MAB292).

### 2.7. Mouse Adipokine Array Kit

Protein adipokine arrays were purchased from R&D systems (Minneapolis, MN, cat number ARY-013) and conducted in accordance with the manufactured protocols. Normalized adipokine levels were calculated as the density of a specific adipokine divided by the density of an internal loading control on each array. Densitometry was calculated with ImageJ PC-based software (National Institute of Health).

### 2.8. Statistical Analysis

All experiments were performed for a minimum of three times. One way analysis of variance (ANOVA), followed by Student-Newman-Keuls Post-Hoc Tests, was performed to determine statistically significant differences between multiple groups. Student's *t*-test was used to determine statistically significant differences between two groups. All statistical tests were run at a 95% confidence interval, and significance was denoted as *P* < 0.05.

## 3. Results and Discussion

### 3.1. Adipocytes Secrete IGF-2 at Levels That Stimulate Breast Cancer Cell Proliferation

**Figure 1:**
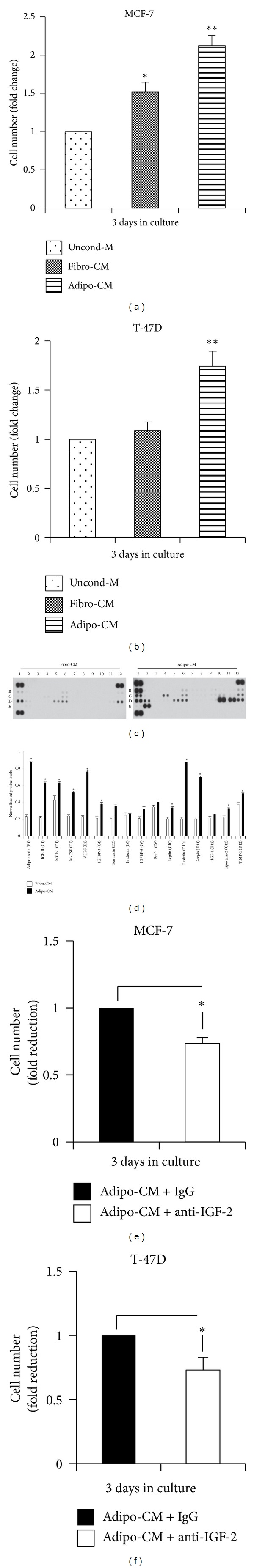
Adipocytes secrete levels of IGF-2 that contribute to adipo-CM stimulated breast cancer cell proliferation. (a, b) The total number of live MCF-7 (a) and T-47D (b) cells grown in unconditioned (uncond-M), fibroblast (fibro-CM), or adipocyte (adipo-CM) conditioned medium for three days in culture was determined and is shown relative to the number of live cells in the uncond-M group, which was arbitrarily assigned a value of 1. Data shown are the means ± S.E. for three replicate experiments, and significant (*P* < 0.05) induction of cell number by fibro-CM (*) compared to the uncond-M group or by adipo-CM (**) compared to the uncond-M and fibro-CM groups is shown. (c, d) Adipokine protein arrays were used to determine the relative levels of adipokines in adipo-CM and fibro-CM. (d) Data shown are the means ± SE for three experiments, and significantly (*P* < 0.05) higher levels of an adipokine in adipo-CM (*) compared to fibro-CM are shown. Normalized adipokine levels were calculated as the densitometry of an adipokine normalized to the densitometry of an internal loading control. (e, f) The total number of live MCF-7 (e) and T-47D (f) cells treated with nonspecific IgG (5 *μ*g/mL) or a specific IGF-2 blocking antibody (5 *μ*g/mL) in adipo-CM for three days in culture was determined and is displayed relative to the number of live cells in the fibro-CM nonspecific IgG group, which was arbitrarily assigned a value of 1. Data shown are the means ± S.E. for three replicate experiments. A significant (*P* < 0.05) decrease in cell number by IGF-2 antibody (*) is shown.

The application of adipocyte conditioned medium (adipo-CM) to MCF-7 or T-47D breast cancer cells for three days in culture significantly (*P* < 0.0001 and *P* < 0.005, resp.) increased proliferation more than the application of fibroblast conditioned medium (fibro-CM) or unconditioned medium (uncond-M) (Figures 1(a) and 1(b)). Adipokine protein arrays revealed that the levels of several adipokines in adipo-CM were significantly higher than in fibro-CM, including insulin-like growth factor-2 (IGF-2) (by approximately 3-fold; (*P* < 0.05)) (Figures 1(c) and 1(d)). Since the levels of IGF-2 were higher in adipo-CM than in fibro-CM (Figures 1(c) and 1(d)), and MCF-7 and T-47D cells overly express IGF-1R and IR-A [11, 12], we questioned the role of IGF-2 in adipo-CM. The addition of a specific IGF-2 blocking antibody to adipo-CM significantly reduced MCF-7 (*P* < 0.0003) and T-47D (*P* < 0.05) proliferation compared to control cells incubated in adipo-CM containing a nonspecific control IgG (Figures 1(e) and 1(f)). This result suggests that adipocyte secreted IGF-2 contributes to the proliferation of ER expressing breast cancer cells.

### 3.2. Ligand-Activated AHR Inhibits Adipo-CM and IGF2 Stimulated Breast Cancer Cell Proliferation

**Figure 2:**
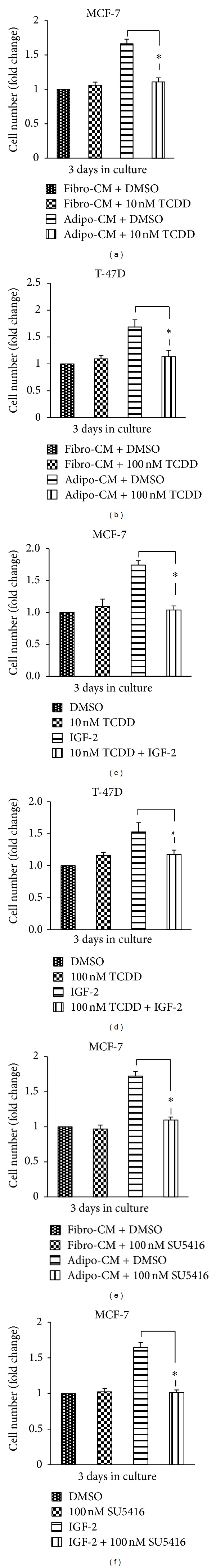
AHR ligands inhibit adipo-CM and IGF-2 stimulated breast cancer cell growth. (a, b) The total number of live MCF-7 (a) and T-47D (b) cells treated with DMSO vehicle or TCDD in fibro-CM or adipo-CM for three days in culture was determined and is displayed relative to the number of live cells in the fibro-CM DMSO control group, which was arbitrarily assigned a value of 1. Data shown are the means ± S.E. for three experiments, and a significant (*P* < 0.05) decrease in cell number by TCDD (*) is indicated. (c, d) The total number of MCF-7 (c) and T47D (d) cells treated with DMSO or TCDD alone or plus IGF-2 (100 ng/mL) for three days in culture was determined and is displayed relative to the number of live cells in the DMSO group, which was assigned a value of 1. Data shown are the means ± S.E. for three experiments, and a significant (*P* < 0.05) decrease in cell number by TCDD (*) is indicated. (e, f) The total number of MCF-7 cells stimulated with DMSO or SU5416 in fibro-CM or adipo-CM (e) or with DMSO or SU5416 alone or plus IGF-2 (100 ng/mL) for three days in culture was determined and is displayed relative to the number of live cells in the DMSO group, which was assigned a value of 1. Data shown are the means ± S.E. for three experiments, and a significant (*P* < 0.05) decrease in cell number by TCDD (*) is indicated.

TCDD is a highly specific AHR agonist [17]. The application of adipo-CM plus TCDD to MCF-7 and T-47D cells significantly (*P* < 0.0001 and *P* < 0.002, resp.) reduced proliferation compared to that observed in control cells stimulated with adipo-CM in the presence of vehicle DMSO (Figures 2(a) and 2(b)). Conversely, MCF-7 and T-47D proliferation in fibro-CM was not inhibited by TCDD (Figures 2(a) and 2(b)). These data suggest that activation of the AHR with a highly specific AHR ligand specifically inhibits the mitogenic effects of adipokines that are present in the adipo-CM.

Next, we conducted experiments to determine whether TCDD inhibits IGF-2 stimulated breast cancer cell proliferation. Treating MCF-7 and T-47D cells with IGF-2 (100 ng/mL) plus TCDD significantly (*P* < 0.0001 and *P* < 0.0034, resp.) inhibited proliferation compared to that observed in control cells stimulated with IGF-2 plus DMSO vehicle (Figures 2(c) and 2(d)). This result indicates that activation of the AHR by a highly specific AHR agonist blocks IGF-2 stimulated breast cancer cell proliferation.

To provide further evidence that ligand-activated AHR inhibits the proliferation effects of adipokines, we tested a second AHR agonist, SU5416 [16]. The application of adipo-CM with SU5416 significantly (*P* < 0.0001) inhibited MCF-7 cell proliferation compared to that observed in adipo-CM plus DMSO vehicle group (Figure 2(e)). SU5416 did not reduce breast cancer cell number in the presence of fibro-CM (Figure 2(e)). Further, SU5416 significantly (*P* < 0.001) blocked IGF-2 stimulated MCF-7 cell proliferation (Figure 2(f)). Collectively, these data indicate that exogenous AHR ligands inhibit the proliferative effects of mitogenic adipokines (including IGF-2) in human ER expressing breast cancer cells.

### 3.3. AHR Ligand-Stimulated Inhibition of IGF-2 Requires the AHR

**Figure 3:**
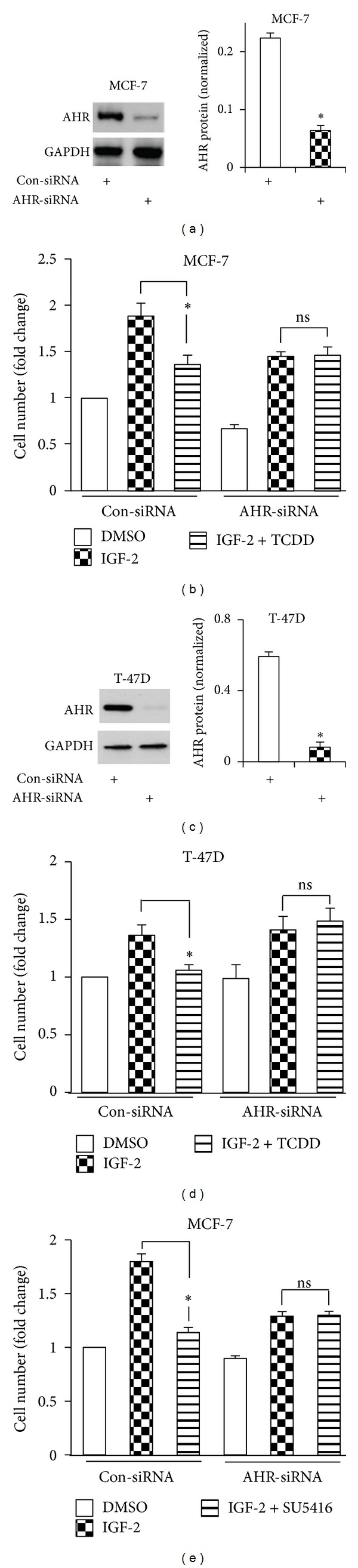
AHR ligand-stimulated inhibition of IGF-2 requires the AHR. (a, c) MCF-7 (a) or T-47D (c) cells transiently transfected with con-siRNA or AHR-siRNA for 36 hr, followed by isolation of total cellular extract and western blot analysis with AHR and GAPDH antibody. GAPDH was used to normalize between samples. Data shown are the means ± S.E. of three experiments. A significant (*P* < 0.05) decrease in AHR by AHR-siRNA (*) is indicated. (b, d) The total number of MCF-7 (b) or T-47D (d) cells transfected with nontargeting control short interfering RNA (con-siRNA) or aryl hydrocarbon receptor siRNA (AHR-siRNA) for 36 hr and then treated with DMSO, vehicle control (con), IGF2 (100 ng/mL) plus DMSO or IGF2 plus TCDD (MCF-7; 10 nM, T-47D; 100 nM) for three additional days in culture was determined and is displayed relative to the number of live cells in the DMSO con-siRNA group, which was assigned a value of 1. Data shown are the means ± S.E. of three experiments. (e) The total number of MCF-7 cells transfected with con-siRNA or AHR-siRNA for 36 hr and then treated with DMSO vehicle control (con), IGF2 (100 ng/mL) plus DMSO, or IGF2 plus SU5416 (100 nM) for three additional days in culture was determined and is displayed relative to the number of live cells in the DMSO con-siRNA group, which was assigned a value of 1. Data shown are the means ± S.E. of three experiments.

Next, experiments were conducted to provide evidence that exogenous AHR ligands inhibit the proliferative effects of adipokines through a mechanism that is dependent on the AHR. To this end, MCF-7 and T-47D cells were transiently transfected with a short interfering RNA that specifically targets the AHR (AHR-siRNA) or with a nontargeting control siRNA (con-siRNA) prior to treatment with IGF-2 (100 ng/mL) supplemented with DMSO vehicle or TCDD. The level of AHR protein in AHR-siRNA transfected MCF-7 and T-47D cells was significantly (*P* < 0.0002 and *P* < 0.0001, resp.) lower than in con-siRNA transfected cells (Figures 3(a) and 3(b)). In the presence of con-siRNA, TCDD significantly (*P* < 0.001) inhibited IGF-2 stimulated MCF-7 proliferation (Figure 3(b)). Reducing the AHR in the presence of IGF-2 significantly (*P* < 0.001) inhibited proliferation relative to control cells treated with con-siRNA plus IGF-2 (Figure 3(b)). This result suggests that in the absence of exogenous AHR ligands, the AHR itself plays an endogenous role in MCF-7 cells that is required for maximal proliferation in the presence of IGF-2. Importantly, as shown in Figure 3(b), reducing AHR prevented TCDD inhibition of IGF-2 (Figure 3(b); compare last two right bars). This latter result provides evidence that TCDD inhibits IGF-2 by activating the AHR, because TCDD does not inhibit IGF-2 in cells with reduced expression of the AHR (Figure 3(b)). The TCDD-stimulated inhibition of IGF-2 by AHR was further confirmed in T-47D cells, which showed the TCDD antiproliferative effects towards IGF-2 were reversed upon AHR knockdown (Figures 3(c) and 3(d)).

To provide evidence that a different AHR ligand also inhibits IGF-2 by specifically activating the AHR, AHR-siRNA experiments were repeated with SU5416. As shown in Figure 3(e), under con-siRNA conditions, SU5416 significantly (*P* < 0.0001) inhibited IGF-2 stimulated MCF-7 cell proliferation. In accordance with our prior result, reducing the AHR itself inhibited MCF-7 cell proliferation in the presence of IGF-2 compared to control cells transfected with con-siRNA and stimulated with IGF-2 (Figure 3(e)). AHR-siRNA blocked SU5416 inhibition of IGF-2 (Figure 3(e); compare last two right bars). Collectively, these data provide mechanistic evidence that upon activation by exogenous AHR agonists TCDD and SU5416, the AHR inhibits IGF-2 stimulated MCF-7 and T-47D proliferation.

### 3.4. TCDD Stimulates Reductions in E2F1, CCND1, MYB, SRC, JAK2, and JUND Gene Expression

To begin to investigate the mechanism by which ligand-activated AHR inhibits proliferation, experiments were conducted to determine whether TCDD reduces the expression of genes that could be important for breast cancer cell proliferation in the presence of IGF-2. To this end, we used a commercially available RT^2^ Profiler PCR Array that is capable of integrating 84 genes that are prooncogenes or tumor suppressors and thus key regulators of tumorigenesis. We focused on comparison between two groups, MCF-7 cells stimulated with IGF-2 and MCF-7 cells cotreated with IGF-2 plus TCDD for 48 hrs. This analysis revealed that the expression of E2F1, CCND1, MYB, SRC, JAK2, and JUND was significantly reduced in cells treated with IGF-2 plus TCDD compared to that observed in cells stimulated with IGF-2 (Table 1; *n* = 3). TCDD inhibition of these specific gene targets could be one mechanism by which ligand-activated AHR inhibits mitogenic adipokine signaling, because the observed downregulated genes in TCDD treated cells play important roles in breast cancer cell proliferation. E2F1 is a transcription factor that binds retinoblastoma protein, enhances the proliferation of human cancer cells, and stimulates increases in the transcription and expression of CCND1 [20]. The tyrosine kinase JAK2 upon activation by cytokine receptor induced signaling to stimulate the phosphorylation and activation of STAT3 [10]. When activated by JAK2, STAT3 promotes the transcription and expression of CCND1 [6]. The basic leucine zipper (bZIP) transcription factor JUND by function as a component of AP-1 enhances the transcription and expression of CCND1 [21]. MYP is 17 beta-estradiol regulated transcription factor that has been reported to be important for the proliferation of ER expressing human breast cancer cells, including T-47D and MCF-7 cells [21].

## 4. Conclusion

In summary, we provide evidence that adipocyte secreted IGF-2 is sufficient to contribute to the proliferation of human ER expressing breast cancer cells. This result suggests that adipocyte secretion of IGF-2 could play a role in promoting breast cancer in the context of obesity. Adipocytes have also been reported to secrete IGF-1 in the presence of high glucose and exogenously added fatty acids [8]. Both IGF-1 and IGF-2 enhance the proliferation of human cancer cells through IGF-1R and IR-A [11, 12]. Using highly specific AHR agonists and AHR-siRNA, we provide the first evidence suggesting that specific ligand activation of the AHR inhibits adipo-CM and IGF-2-stimulated proliferation of ER positive human breast cancer cells. It is important to note that E2F1, SCR, JAK2, and JUND are critical upstream transcriptional regulators of CCND1 [6, 10, 20, 22]. Thus, their downregulation by TCDD (Table 1) could be one mechanism by which TCDD downregulates CCND1 expression. CCND1 is also a critical mediator of the proliferative effects of IGF proteins and other adipokines like leptin [6, 12]. Therefore, TCDD inhibition of CCND1 expression (Table 1) is likely to be one of the major mechanisms that inhibit mitogenic adipokine signaling in breast cancer cells. The results of this study provide the impetus for future study investigating the transcriptional mechanisms by which ligand-activated AHR by regulating the expression of prooncogenes modulates mitogenic adipokine signaling in human breast cancer cells. Collectively, this report provides evidence that drugs that target the AHR may reduce breast cancer risk in the context of human obesity.

## Figures and Tables

**Table 1:** Gene expression profiling shows significantly (*P* < 0.05) reduced expression of proto-oncogenes in MCF-7 cells stimulated with IGF-2 plus TCDD compared to cells stimulated with IGF-2 at 48 hrs after treatment (*n* = 3).

Symbol	Description	*P* value	Fold regulation
E2F1	E2F transcription factor 1	0.042684	−4.0169
CCND1	Cyclin D1	0.007724	−3.1767
MYB	V-myb myeloblastosis viral oncogene homolog (avian)	0.026054	−3.1760
SRC	V-src sarcoma (Schmidt-Ruppin A-2) viral oncogene homolog (avian)	0.027237	−2.5269
JAK2	Janus kinase 2	0.028474	−2.0015
JUND	Jun D proto-oncogene	0.026855	−2.0066

## References

[1] Calle EE (2007). Obesity and cancer. *British Medical Journal*.

[2] Calle EE, Kaaks R (2004). Overweight, obesity and cancer: Epidemiological evidence and proposed mechanisms. *Nature Reviews Cancer*.

[3] Khandekar MJ, Cohen P, Spiegelman BM (2011). Molecular mechanisms of cancer development in obesity. *Nature Reviews Cancer*.

[4] Iyengar P, Combs TP, Shah SJ (2003). Adipocyte-secreted factors synergistically promote mammary tumorigenesis through induction of anti-apoptotic transcriptional programs and proto-oncogene stabilization. *Oncogene*.

[5] Iyengar P, Espina V, Williams TW (2005). Adipocyte-derived collagen VI affects early mammary tumor progression in vivo, demonstrating a critical interaction in the tumor/stroma microenvironment. *Journal of Clinical Investigation*.

[6] Saxena NK, Vertino PM, Anania FA, Sharma D (2007). Leptin-induced growth stimulation of breast cancer cells involves recruitment of histone acetyltransferases and mediator complex to CYCLIN D1 promoter via activation of Stat3. *Journal of Biological Chemistry*.

[7] Hu X, Juneja SC, Maihle NJ, Cleary MP (2002). Leptin: a growth factor in normal and malignant breast cells and for normal mammary gland development. *Journal of the National Cancer Institute*.

[8] D'Esposito V, Passaretti F, Hammarstedt A (2012). Adipocyte-released insulin-like growth factor-1 is regulated by glucose and fatty acids and controls breast cancer cell growth in vitro. *Diabetologia*.

[9] Vona-Davis L, Rose DP (2007). Adipokines as endocrine, paracrine, and autocrine factors in breast cancer risk and progression. *Endocrine-Related Cancer*.

[10] Bahrenberg G, Behrmann I, Barthel A (2002). Identification of the critical sequence elements in the cytoplasmic domain of leptin receptor isoforms required for Janus kinase/signal transducer and activator of transcription activation by receptor heterodimers. *Molecular Endocrinology*.

[11] Cohen DH, LeRoith D (2012). Obesity, type 2 diabetes, and cancer: the insulin and IGF connection. *Endocrine-Related Cancer*.

[12] Gallagher EJ, LeRoith D (2011). Minireview: IGF, insulin, and cancer. *Endocrinology*.

[13] Musgrove EA, Caldon CE, Barraclough J, Stone A, Sutherland RL (2011). Cyclin D as a therapeutic target in cancer. *Nature Reviews Cancer*.

[14] Safe S, Qin C, McDougal A (1999). Development of selective aryl hydrocarbon receptor modulators for treatment of breast cancer. *Expert Opinion on Investigational Drugs*.

[15] Zhang S, Lei P, Liu X (2009). The aryl hydrocarbon receptor as a target for estrogen receptor-negative breast cancer chemotherapy. *Endocrine-Related Cancer*.

[16] Mezrich JD, Nguyen LP, Kennedy G (2012). SU5416, a VEGF receptor inhibitor and ligand of the AHR, represents a new alternative for immunomodulation. *PloS ONE*.

[17] Denison MS, Soshilov AA, He G, Degroot DE, Zhao B (2011). Exactly the same but different: promiscuity and diversity in the molecular mechanisms of action of the aryl hydrocarbon (dioxin) receptor. *Toxicological Sciences*.

[18] Sparano JA, Wang M, Zhao F (2012). Obesity at diagnosis is associated with inferior outcomes in hormone receptor-positive operable breast cancer. *Cancer*.

[19] Worster DT, Schmelzle T, Solimini NL (2012). Akt and ERK control the proliferative response of mammary epithelial cells to the growth factors IGF-1 and EGF through the cell cycle inhibitor p57Kip2. *Science Signaling*.

[20] Lee RJ, Albanese C, Fu M (2000). Cyclin D1 is required for transformation by activated Neu and is induced through an E2F-dependent signaling pathway. *Molecular and Cellular Biology*.

[21] Toualbi-Abed K, Daniel F, Güller MC (2008). Jun D cooperates with p65 to activate the proximal *κ*B site of the cyclin D1 promoter: role of PI3K/PDK-1. *Carcinogenesis*.

[22] Drabsch Y, Hugo H, Zhang R (2007). Mechanism of and requirement for estrogen-regulated MYB expression in estrogen-receptor-positive breast cancer cells. *Proceedings of the National Academy of Sciences of the United States of America*.

